# Correlation exploration of metabolic and genomic diversity in rice

**DOI:** 10.1186/1471-2164-10-568

**Published:** 2009-12-01

**Authors:** Keiichi Mochida, Taku Furuta, Kaworu Ebana, Kazuo Shinozaki, Jun Kikuchi

**Affiliations:** 1 RIKEN Plant Science Center, 1-7-22, Suehiro-cho, Tsurumi, Yokohama 230-0045, Japan; 2 Graduate School of BionanoSciences, Yokohama City University, 1-7-29, Suehiro-cho, Tsurumi, Yokohama 230-0045, Japan; 3 National Institute of Agrobiological Sciences, 2-1-2, Kannondai, Tsukuba, 305-8602 Japan; 4 Graduate School of Bioagriculture Sciences, Nagoya University, 1-1 Furo-cho, Chikusa-ku, Nagoya 464-8601, Japan

## Abstract

**Background:**

It is essential to elucidate the relationship between metabolic and genomic diversity to understand the genetic regulatory networks associated with the changing metabolo-phenotype among natural variation and/or populations. Recent innovations in metabolomics technologies allow us to grasp the comprehensive features of the metabolome. Metabolite quantitative trait analysis is a key approach for the identification of genetic loci involved in metabolite variation using segregated populations. Although several attempts have been made to find correlative relationships between genetic and metabolic diversity among natural populations in various organisms, it is still unclear whether it is possible to discover such correlations between each metabolite and the polymorphisms found at each chromosomal location. To assess the correlative relationship between the metabolic and genomic diversity found in rice accessions, we compared the distance matrices for these two "omics" patterns in the rice accessions.

**Results:**

We selected 18 accessions from the world rice collection based on their population structure. To determine the genomic diversity of the rice genome, we genotyped 128 restriction fragment length polymorphism (RFLP) markers to calculate the genetic distance among the accessions. To identify the variations in the metabolic fingerprint, a soluble extract from the seed grain of each accession was analyzed with one dimensional ^1^H-nuclear magnetic resonance (NMR). We found no correlation between global metabolic diversity and the phylogenetic relationships among the rice accessions (*r*_s _= 0.14) by analyzing the distance matrices (calculated from the pattern of the metabolic fingerprint in the 4.29- to 0.71-ppm ^1^H chemical shift) and the genetic distance on the basis of the RFLP markers. However, local correlation analysis between the distance matrices (derived from each 0.04-ppm integral region of the ^1^H chemical shift) against genetic distance matrices (derived from sets of 3 adjacent markers along each chromosome), generated clear correlations (*r*_s _> 0.4, p < 0.001) at 34 RFLP markers.

**Conclusion:**

This combinatorial approach will be valuable for exploring the correlative relationships between metabolic and genomic diversity. It will facilitate the elucidation of complex regulatory networks and those of evolutionary significance in plant metabolic systems.

## Background

It is essential to elucidate the relationships between metabolic and genomic diversity to understand the genetic causes of phenotypic variation among natural populations. Visible and chemical variations associated with genomic diversity provide the information required to identify key genes associated with such phenotypic changes [1]. Patterns of nucleotide polymorphisms and a population structure figured in a natural population have allowed us to understand the evolutionary significance of genetic variation under the influence of geographic factors or different environments [2,3].

The technical development of metabolite profiling has provided quantitative phenotypic information on the metabolome among strains and identified genes involved in metabolic networks [4]. Therefore, a comprehensive exploration of the correlations between metabolic and genomic diversity, achieved by superimposing data from these two "omics" approaches, could provide information regarding both the broad and specific relationships between metabolo-phenotypes and genotypes. This information would aid the identification of genetic associations between the metabolic and/or visible phenotypes [5,6].

Metabolite quantitative trait loci (mQTLs) analysis using segregated populations has been applied to various plant species as a popular forward genetics approach [4,5,7-9]. Although mQTLs analysis has been used to dissect the genetic loci involved in metabolo-phenotypic changes in mapping populations of various organisms, no methods have been developed to identify the correlations between genetic polymorphisms and metabolo-phenotypic differences in divergent accessions or individuals from natural populations. To date, several attempts have been performed to find correlative relationships between genetic and metabolic diversity in various organisms [10-13]. It is still unclear whether it is possible to discover such correlations between each metabolite and the polymorphic pattern found at each chromosomal location to explain natural variation. Combinatorial approaches of population genomics coupled with metabolo-phenotyping should play a significant role in the exploration of the association of genetic variation with metabolic changes that have a significant impact on evolution and adaptation [3].

In this study, to assess the correlative relationship between metabolic and genomic diversity found in 18 rice accessions selected from the world rice collection, we compared the distance relationships of accessions for these two omics patterns, globally, with marker polymorphisms vs. the metabolomic fingerprints of NMR spectra, as well as locally, for each chromosomal region vs. each spectra. To perform these analyses, we developed a robust procedure to explore the relationship between metabolic and genomic diversity. As a result, we present the first correlation map of metabolic and genomic diversity among rice accessions.

## Results and Discussion

**Figure 1:**
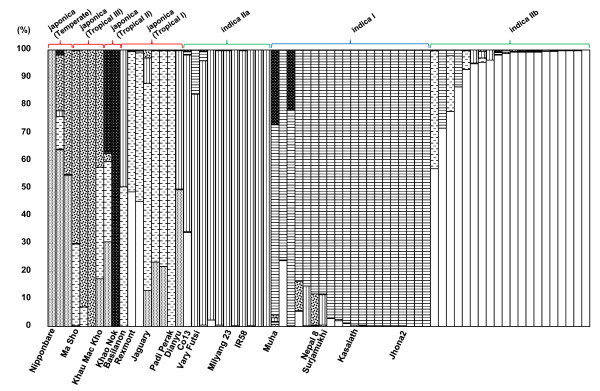
**Population structure of 68 rice accessions from around the world (including the 18 used in this study) based on the genotype data of 128 RFLP markers determined with Structure software**.

To select the appropriate rice accessions for the comparison of metabolic profiles with patterns of genetic polymorphisms, we initially investigated the population structure of the world rice collection. The accessions of the world rice collection have been classified into three major groups, japonica, indica I, and indica II [14]. To determine in greater detail the genetic structure of these populations, we performed population structure analysis using Structure software on data generated by genotyping 128 restriction-fragment-length polymorphism (RFLP) markers. This analysis showed that the japonica type accessions were composed of 4 sub-groups; temperate, and tropical I, II, and III. For the twoindica groups, the members of indica I were not divided, while those of indica II were divided into two sub-groups; indica IIa and indica IIb (Fig. 1). We chose 18 rice accessions from 6 representative sub-groups to cover a diverse range of cultivated rice populations, on the basis of population structure, to avoid regional bias (Fig. 1 and Additional file 1).

**Figure 2:**
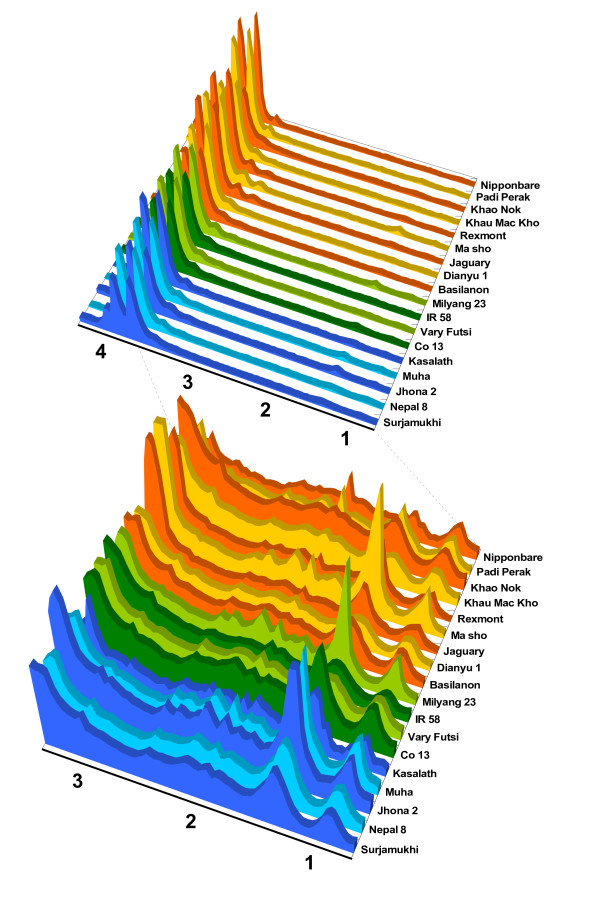
**Diversity of soluble metabolites among the 18 rice accessions**. The global metabolic profiles of soluble metabolites were determined from 0.04-ppm integral values based on ^1^H NMR spectra. The 1D ^1^H NMR spectra in the range of chemical shifts from 4.29 to 0.71 ppm are shown, along with a close-up of the spectra from 3.3 ppm. Orange, japonica; blue, indica I; green, indica II.

The RFLP genotyping data were also used to generate global and local genetic distance matrices along each chromosome in combination with physical allocation data on the rice genome (Additional file 2). Soluble extracts of seeds from each accession were analyzed by 1D ^1^H-nuclear magnetic resonance (NMR) analysis to elucidate the metabolic diversity among the accessions (Fig. 2 and Additional file 3).

**Figure 3:**
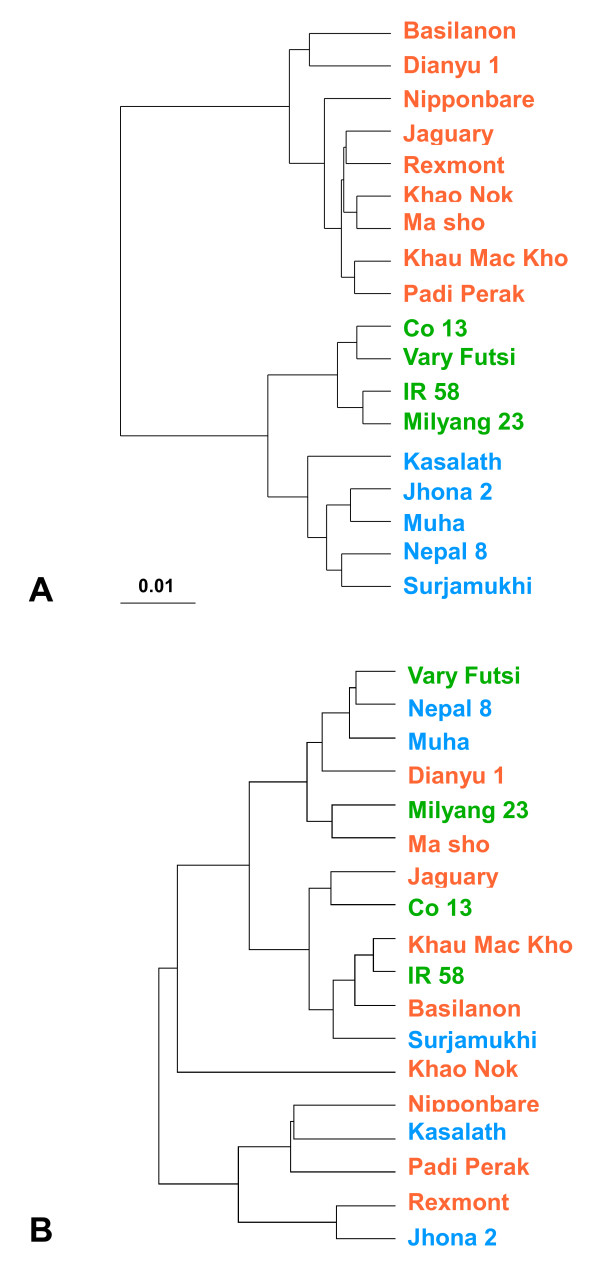
**Global genomic and metabolo-phenotypic diversity among the 18 rice accessions**. Spearman's coefficient of correlation between the genetic and Euclidian distance matrices of the 1D NMR data was calculated. (A) Dendrogram of the phylogenetic tree obtained by the UPGMA method. (B) Metabolomic distance based on normalized data from the integral regions of the ^1^H NMR spectra is shown in Additional file 3 Table S2.

To assess the global correlation between metabolic and genomic diversity, we calculated Spearman's coefficient of correlation (*r*_s_) between the distance matrices calculated from the 4.29- to 0.71-ppm ^1^H chemical shifts and genetic distance among accessions with polymorphisms at the 128 RFLP markers. We found no correlation between global metabolic diversity and the phylogenetic relationships among the rice accessions (*r*_s _= 0.14). Although the phylogenetic tree of the genetic distance matrix showed the expected 3 typical clusters (corresponding to japonica, indica I, and indica II; Fig. 3a), the metabolic diversity tree showed no identifiable clusters corresponding to the phylogenetic clusters (Fig. 3b). This result indicates that there are no relationships between the genomic and metabolic diversity of solution metabolites of rice grain in the comparison of holistic data. In higher plants, the diversity of the metabolome might tend to be unrelated with genomic diversity. In sesame, which has been an example for the comparison of metabolic and genomic diversity in higher plants, the relationship patterns generated for the AFLPs and the seed metabolic profiles also differed [10]. Seed metabolic profiles are derived from the transcriptome that is specifically activated in the embryo and endosperm during seed development. Genes involved in the abundance of solution metabolites of rice seed might consist of small members expressed in seed specific transcriptome. Although there have been few examples of the global assessment of correlations between metabolic and genomic diversity in higher plants, the recent updating of the infrastructure for population genomics and metabolomics should allow us to determine correlation patterns at different levels i.e., from genomic segments to small chromosomal regions in each organism along with their evolutionary patterns. In *Arabidopsis*, a large number of metabolic QTLs were identified in a recombinant inbred line (RIL) of two parental ecotypes, which had been selected from 14 ecotypes based on their metabolic profiles [15]. Most of the metabolic variation observed among the ecotypes was successfully mapped using this RIL population, indicating that metabolic variation can be associated with simplified polymorphic patterns at specific chromosomal regions.

**Figure 4:**
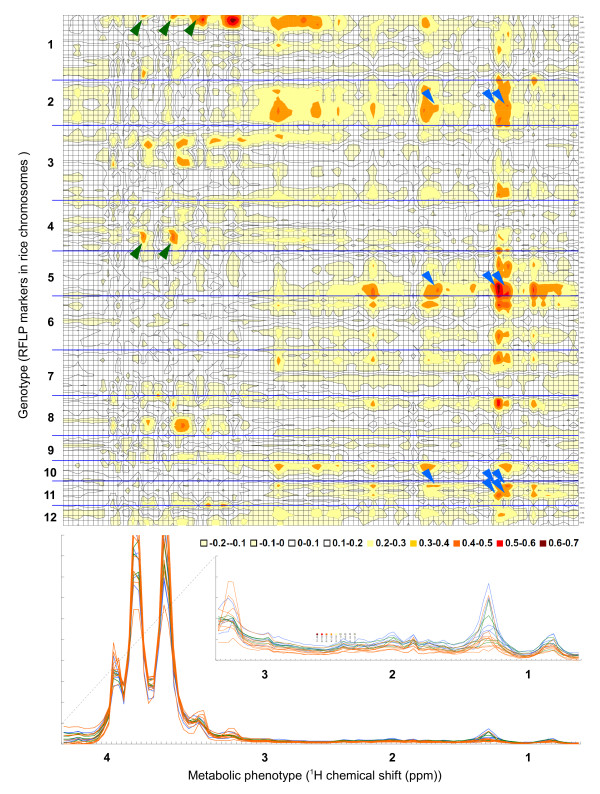
**Correlation display between genomic and metabolic diversity in rice. **Top: Heatmap displaying coefficients of correlation between metabolite abundance based on the ^1^H chemical shift (horizontal) and polymorphisms along each of the 12 rice chromosomes (vertical) in the 18 rice accessions. Bottom: Overlay of ^1^H-NMR spectra of metabolite extracts from the 18 rice accessions. Orange, japonica; blue, indica I; green, indica II. Intra-molecular correlations against more than two NMR signals can be determined; these are allocated on the chromosomal regions in this correlation display (green and blue arrows for sucrose and lipids, respectively).

We then calculated the *r*_s _of Euclidian distance matrices derived from each 0.04-ppm integral region of the ^1^H chemical shift against the genetic distance matrices derived from sets of three adjacent markers along each chromosome (Additional file 4). The results of these local analyses are presented as a 2D correlation map (Fig. 4). To assess the statistical significance of each correlation coefficient value in this dataset, we calculated the p-value for each *r*_s _value and determined the relationship between the *r*_s _value and the p-value for all combinations. As a result of this analysis, an *r*_s _value of 0.4 was supported with a p-value of 2.99E-07; the value of *r*_s _at the 0.1% level of the p-value was between ≥ 0.3 and ≤ 0.4. We also calculated the correlation coefficient values between each genetic distance matrix of three randomly sampled RFLP markers and the metabolic profile of each of the 104 NMR spectra in 10,000 trials to estimate the probability of a high correlation by random chance (Additional file 5). The results showed that the probability of a correlation coefficient *r*_s _≥ 0.4 in the randomized data was less than 1% (0.71%). Therefore, in this analysis, we applied *r*_s _= 0.4 as the threshold to detect correlative combinations between metabolic and genomic diversity.

The presentation of the correlations as a 2D heat map showed large non-correlative areas studded with correlative areas, which can be explored in omics-wide comparisons. The heat map showed clear correlations between the profiles of soluble metabolites and local genomic diversity at each chromosomal location. To identify metabolites, we performed 2D ^1^H-^13^C NMR analysis (Additional file 6, 7 and 8). Diversity in the choline profile (3.2 ppm) showed a high correlation with genetic diversity around the R1613 RFLP marker, located at 176.5 cM on chromosome (Chr.) 1 (*r*_s _= 0.67, *P *< 0.01). The lipid methylene signal (1.22 ppm) showed correlations with polymorphic sites at multiple chromosomal locations, with the highest located around the C43 RFLP marker at 92.8 cM on Chr. 5 (*r*_s _= 0.66, *P *< 0.01), and the second highest around R1943 at 123.7 cM on Chr. 8 (*r*_s _= 0.58, *P *< 0.01). The correlation peaks corresponding to each of the lipids (1.00, 1.16, 1.22, and 1.60 ppm) with Chr. 2 (153.6 and 95.7 cM), Chr. 5 (41.4, 92.8, 103.5, 112.3, and 9.8 cM), and Chr. 11 (123.3 and 85.8 cM) were in good accordance with the covalent bond networks within molecules of each compound. Furthermore, sucrose (3.40, 3.59, and 3.78 ppm) with Chr. 1 (186.8 cM) and Chr. 4 (31.4 cM) also showed good accordance. Similar intramolecular covalent bond networks were reported in NMR experiments of biological metabolite mixtures, including covariance total correlation spectroscopy (TOCSY) analysis of insect venom [16] and TOCSY analysis of intact human gut biopsies [17].

Local comparison between the dendrogram based on the correlative RFLP polymorphisms and NMR spectra showed a similar tree structure between both genetic and metabolic variation among the accessions. For example, the dendrogram of genetic polymorphisms around the C43 RFLP marker (92.8 cM on Chr. 5) and the diversity of the lipid methylene signal indicated the genetic and metabolic divergence between indica I and two other groups, japonica and indica II (Additional file 9).

To display the chromosomal locations of the correlative regions at 34 RFLP markers, the results of the correlation analysis were superimposed onto a genetic map of rice (Additional file 10). This genetic map should allow us to identify metabolic QTLs associated with each of the metabolic abundances allocated on a genetic map. It would be beneficial if future genetic analyses narrowed down candidate genes and/or isolated such metabolic QTLs in combination with other genomic resources for rice such as genetic marker systems and genome annotation databases.

Throughout this study, we developed a robust approach to explore the correlation between genomic and metabolomic diversity observed in natural variation, which is shown as a schematic flow in additional file 11. The procedure is summarized as follows: (1) selection of appropriate accessions based on population structures; (2) acquisition of the metabolic profile of selected accessions using various types of metabolomics platforms and the calculation of the distance among accessions on the basis of metabolite abundance or spectrum intensities; (3) genotyping of selected accessions using DNA markers covering the whole genome and the calculation of local genetic distance among accessions from genotype data of adjacent marker sets; (4) to discover the correlative relationships between each metabolite and chromosomal region and the coefficients of correlation for metabolic and genetic distances in all-against-all combinations and to display their distribution on a genetic map. In this analysis of rice, we did not find a correlation between metabolic and genomic diversity as observed in previously reported studies. On the other hand, local correlation analysis allowed us to identify small correlative areas mostly in non-correlative spaces. This suggests that local correlation analysis could be an effective method to discover such correlative areas allocated to chromosomal regions together with linkage maps or variation data of genomic sequences [18,19].

This study illustrated a new integrative approach to explore the links between metabolomics and genomics. The procedure could be extended to a higher resolution by using denser polymorphic marker maps in various species.

## Conclusion

This study assessed the correlative relationships between genetic and metabolic diversity among rice accessions. The data presents the first correlative relationship for the chromosomal distribution of each metabolic profile in rice. The combinatorial use of genomic and metabolomic data demonstrated here should facilitate the elucidation of complex regulatory networks and those of evolutionary significance in plant metabolic systems.

## Methods

### Rice seed materials

The rice accessions were chosen from the RFLP-based Rice Discovery Research Set of rice germplasms, which contains 68 rice accessions and is developed and maintained by Genebank http://www.gene.affrc.go.jp/about_en.php at the National Institute of Agrobiological Sciences (NIAS). The accessions were classified into three major groups by principal components analysis (PCA) of the RFLP data: japonica, indica I, and indica II [14]. Population structure analysis using Structure software (v. 2.1, http://pritch.bsd.uchicago.edu/software/structure2_1.html) was also conducted on the world rice collection to determine the population structure in more detail, especially for the japonica type [20,21]. Based on the results of the PCA and Structure analyses we selected nine accessions each of japonica (temperate, tropical I, II, and III) and indica (indica I and indica II) to be included in the correlation analysis between genetic and metabolomic diversity.

### RFLP genotyping and phylogenetic analysis of rice accessions

We used 128 RFLP markers to evaluate the genetic diversity of the 18 accessions by the Southern hybridization method of Kojima et al. (2005). Each of the marker alleles was scored as Nipponbare-type (1), Kasalath-type (2), or other types (3-7) (Additional file 3), and the presence or absence of each allele was recorded. The genetic distances between the 18 rice accessions were calculated with the "restdist" program of the PHYLIP (v. 3.67) package http://evolution.genetics.washington.edu/phylip.html; we used the distance matrix to calculate the coefficients of correlation with the metabolic diversity matrix and to calculate a phylogenetic tree by the UPGMA method using the "neighbor" program of PHYLIP. The physical position of each RFLP marker was retrieved from the Rice Annotation Project Database (RAP-DB) http://rapdb.dna.affrc.go.jp/[19].

### NMR spectroscopy and quantitative analysis

We prepared NMR samples from rice seeds, as previously described [22,23] with slight modifications. All 1D Watergate [24] spectra were acquired at 298 K on a Bruker Avance DRX 500 NMR spectrometer operating at 500.13 MHz and equipped with a ^1^H inverse triple-resonance probe with triple-axis gradients.

The 1D NMR spectra were integrated between 0.0 and 10.0 ppm over a series of 0.04-ppm regions by our custom integration software [25]. After exclusion of the water and DSS (2,2-dimethyl-2-silapentane-5-sulfonate) resonances, each integral region was normalized to the total integral region (Additional file 2). The data were analyzed by a partial least-squares projection, based on the spectral bins obtained from 1D spectral analysis and by using a hierarchical clustering analysis package running on R software.

### Correlation display of genetic and metabolic variation

A Euclidian distance matrix based on the 0.04-ppm integral regions of the chemical shift from 0.708 to 4.292 ppm (104 bins) in the 1D NMR spectra was calculated by using the "dist" command of the R package. Genetic diversity among the 18 rice accessions was calculated from the genotype data of all alleles of the 128 scored RFLP markers. To assess the correlation between genome- and metabolome-wide diversity among the 18 accessions, we calculated Spearman's coefficient of correlation between the genetic and Euclidian distance matrices. Local metabolic diversity among the 18 accessions was calculated as a Euclidian distance matrix in each of the 104 bins. Local genomic diversity was also calculated as a genetic distance matrix using sliding bins that included three adjacent RFLP markers along each rice chromosome. To explore the local correlations between genomic and metabolic diversity, we calculated Spearman's *r*_s _and p-values for all combinations of the local metabolic and genomic distance matrices using the "cor.test" program of the R package (Additional file 4). An RFLP map with correlative chromosomal positions was generated with MapChart ver. 2.2 [26].

### Annotation of candidate metabolites by 2D NMR of ^13^C-labelled rice seeds

^13^C-labelled rice extracts were prepared as previously described methods with minor modifications [27,28]. In brief, seeds were powdered, 20 mg of which was suspended in 600 μL standard buffer (100 mM potassium phosphate, pH 7.0, and 1.0 mM 2,2-dimethyl-2-silapentane-5-sulfonate in D_2_O), heated at 65°C for 15 min, and centrifuged at 12,000 × *g *for 5 min. The supernatant (500 μL) was decanted into a 5 mm diameter NMR tube.

Two-dimensional (2D) heteronuclear single quantum coherence (HSQC) [29] spectra were recorded on a Bruker Avance 500 spectrometer equipped with an inverse triple resonance CryoProbe with a *Z*-axis gradient, operating at 500.13 MHz for ^1^H frequency (176.061 MHz for ^13^C frequency), and the temperature of the NMR samples was maintained at 298 K. A total of 128 complex f1 (^13^C) and 1024 complex f2 (^1^H) points were recorded with 96 scans per f1 increment, resulting in a total recording time of about 6 h. The spectral window and offset frequency in the f1 dimension were 7042.593 Hz (40 ppm) and 11971.59 Hz (68 ppm), respectively. The spectral window in the f2 dimension was 11160.7 Hz (16 ppm). The offset frequency in the f2 dimension was 3330 Hz (4.75 ppm).

The 2D HSQC spectra of the ^13^C-labelled rice samples were processed using the NMRPipe software package [30]. To quantify the signal intensities, a Lorentzian-to-Gaussian window with a Lorentzian line width of 10 Hz and a Gaussian line width of 15 Hz was applied in both dimensions before Fourier transformation. An automatic polynomial baseline correction was subsequently applied in the f1 dimension. The indirect dimension was zero-filled to 4096 points in the final data matrix. Cross peaks of each metabolite were matched to our standard ^1^H/^13^C chemical shift database [31]. The database is implemented with an in-house Java program, which allows for the systematic batch identification of large numbers of metabolites by simply matching the observed ^13^C-HSQC peaks with peaks in the database. Our chemical shift data are continuously updated and are available on the PRIMe website http://prime.psc.riken.jp/[32]. The queried peaks were classified as annotated on the basis of whether the chemical shift difference in each dimension between the observed peak and the peak in the database was less than a specified tolerance value. Typically, tolerances of 0.03 ppm for ^1^H and 0.53 ppm for ^13^C were used in this study (Additional file 8). From these candidate metabolites, an identification or assignment was defined as unique if there was only one candidate in the database within the specified tolerances for an observed peak (Additional file 6).

## Authors' contributions

KM and JK designed the project, performed data analysis, and drafted the manuscript. TF performed NMR data processing and analyses. EK provided RFLP polymorphism data and population structure of the rice core collection. KS served as the principal investigator of the project. All the authors have contributed to writing this manuscript and have read and approved the contents of the final submitted version.

## Supplementary Material

Additional file 1 **Figure S1**. Rice seed material used in this study and their origins. Click here for file

Additional file 2 **Table S1**. The scored genotype data from the 128 RFLP markers. Click here for file

Additional file 3 **Table S2**. The numeric metabolite profiling data from rice seed extract based on the ^1^H NMR spectra. Click here for file

Additional file 4 **Table S3**. The coefficients of correlation values between genetic and metabolic diversity in rice. Click here for file

Additional file 5 **Figure S2**. Histogram of the correlation coefficient for the randomized data from 10,000 trials. Click here for file

Additional file 6 **Table S4**. The data of the annotated metabolites. Click here for file

Additional file 7 **Figure S3**. The ^13^C-HSQC spectra of seed extracts from the japonica and indica rice varieties. Click here for file

Additional file 8 **Figure S4**. The ^13^C-HSQC spectrum from a seed extract of ^13^C-labelled Nipponbare. Click here for file

Additional file 9 **Figure S5**. The correlative local genetic and metabolo-phenotypic diversity in rice. Click here for file

Additional file 10 **Figure S6**. The correlative chromosomal regions for each NMR spectrum allocated on an RFLP map of rice. Click here for file

Additional file 11 **Figure S7**. A schematic representation of the work flow used to explore the correlations between metabolic and genomic diversity with genetic resources. Click here for file
