# Supplementary material for: Correlation exploration of metabolic and genomic diversity in rice
Source: BMC Genomics. 2009 Dec 1;10:568. doi: 10.1186/1471-2164-10-568 (PMC3087559; doi:10.1186/1471-2164-10-568)
Supplement: Additional file 1 — Figure S1. Rice seed material used in this study and their origins. [file 1471-2164-10-568-S1.PDF]

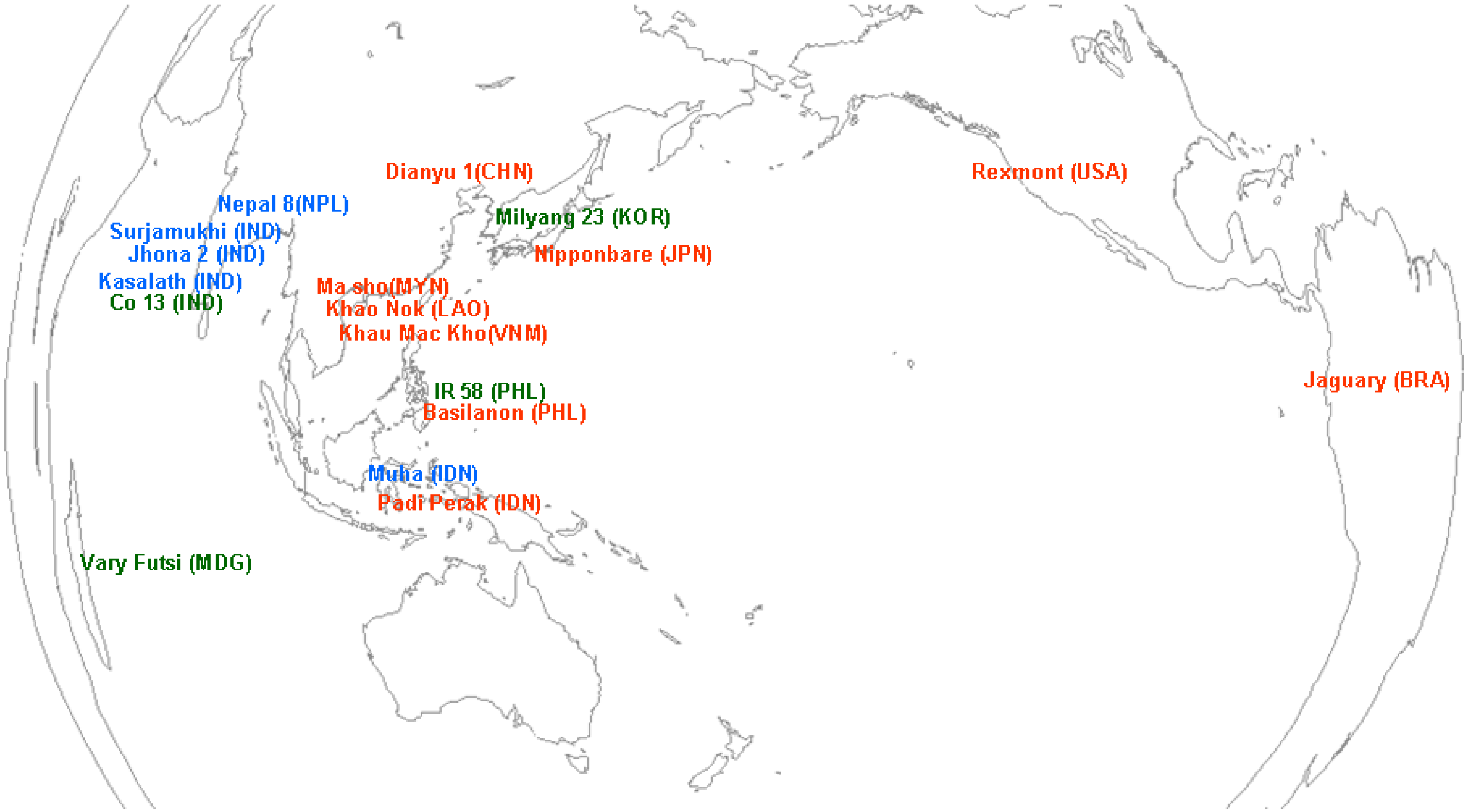

A

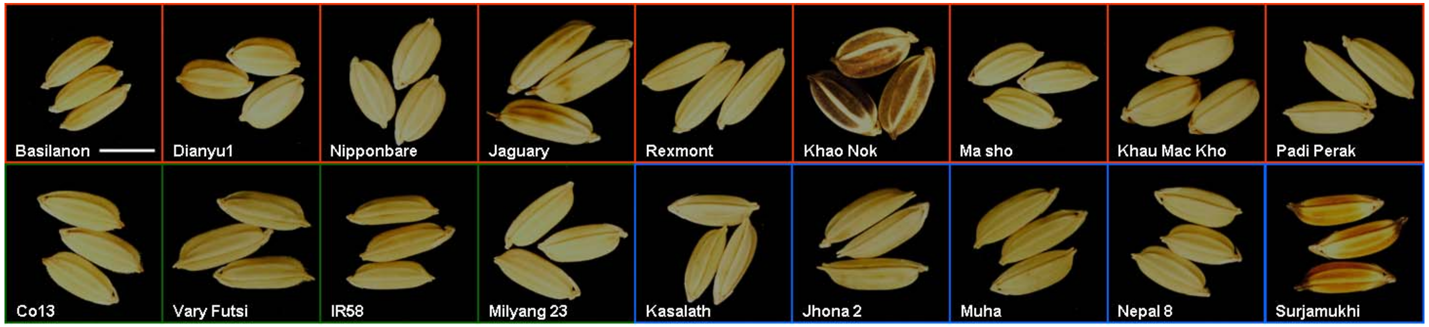

B

Figure. S1 Rice seed materials used in this analysis (B) and their origins (A): orange, japonica type; blue, indica I; green: indica II.
