# Supplementary material for: Correlation exploration of metabolic and genomic diversity in rice
Source: BMC Genomics. 2009 Dec 1;10:568. doi: 10.1186/1471-2164-10-568 (PMC3087559; doi:10.1186/1471-2164-10-568)
Supplement: Additional file 5 — Figure S2. Histogram of the correlation coefficient for the randomized data from 10,000 trials. [file 1471-2164-10-568-S5.PDF]

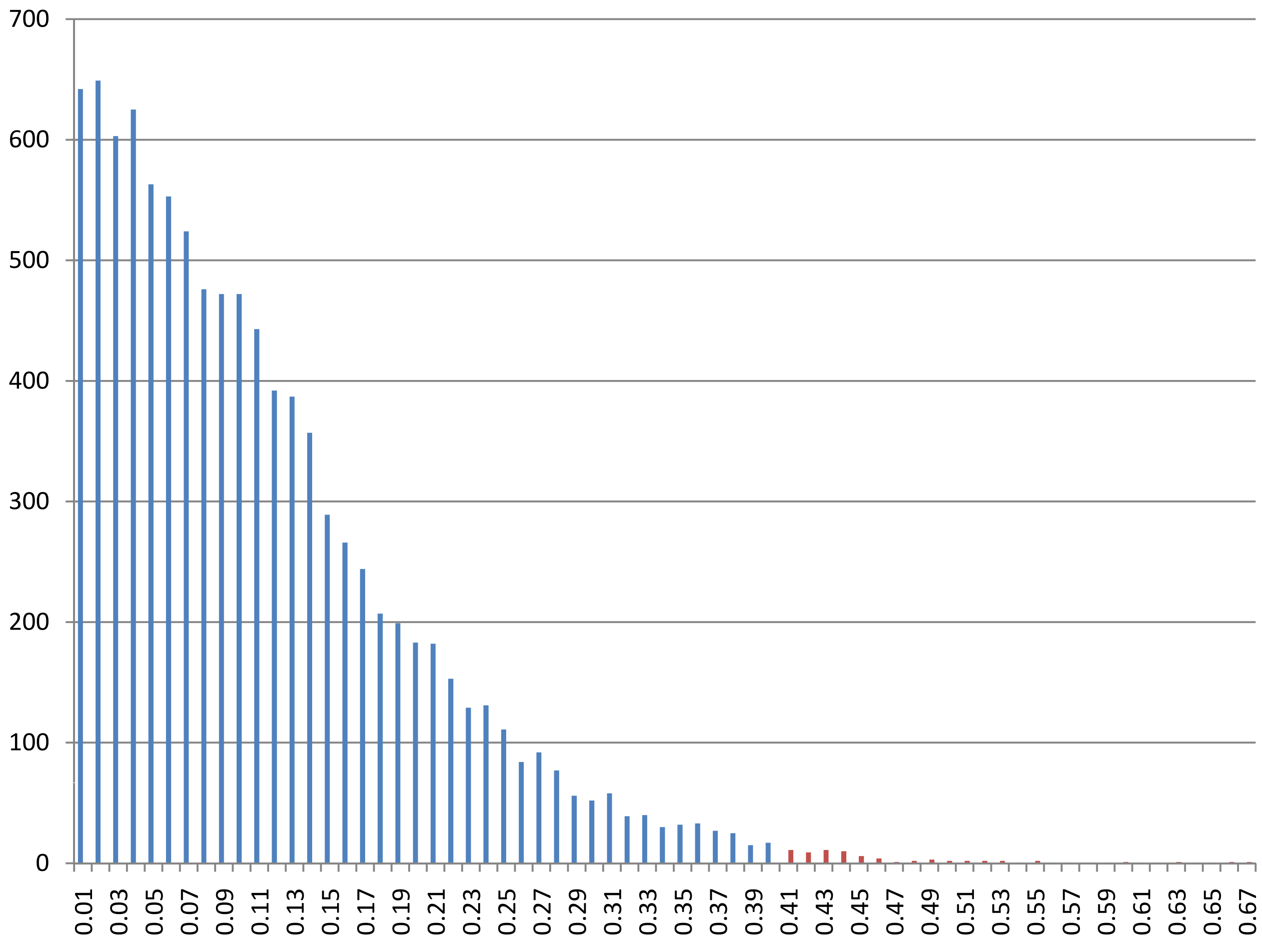

Figure S2. The histogram of correlation coefficient in the randomized data from 10,000 trials. A random sampling test showed 71 cases with r_s>=0.4 in 10,000 trials (Red bars).
