# Supplementary material for: Correlation exploration of metabolic and genomic diversity in rice
Source: BMC Genomics. 2009 Dec 1;10:568. doi: 10.1186/1471-2164-10-568 (PMC3087559; doi:10.1186/1471-2164-10-568)
Supplement: Additional file 8 — Figure S4. The 13C-HSQC spectrum from a seed extract of 13C-labelled Nipponbare. [file 1471-2164-10-568-S8.PDF]

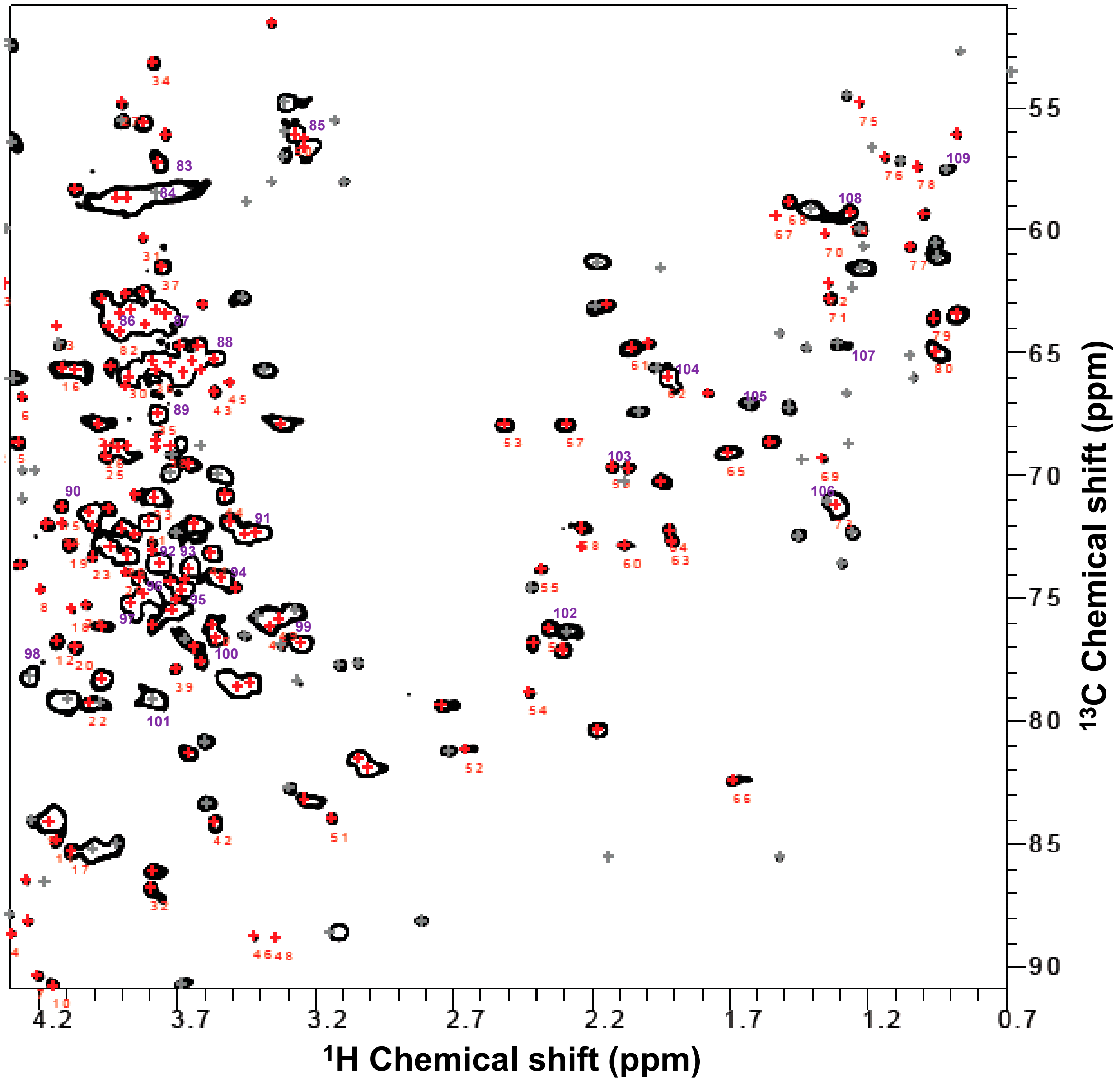

Figure S4. To annotate the metabolite peaks in the rice seed extracts measured by ^{1}H-NMR and 2D ^{13}C-HSQC, ^{13}C-labelled rice was analysed to provide spectral data of the soluble metabolites in order to identify candidate compounds. In total, 189 metabolites were assigned to possible candidate compounds in the metabolite chemical shift database on the PRIMe website. Those candidate metabolites corresponding to each of the bins of ^{1}H NMR chemical shift data are listed in Table S4. A red "+" indicates a metabolite peak assigned to a candidate metabolite listed in the PRIMe database. A grey "+" indicates an anonymous metabolite peak. Peak IDs coloured red indicate unique assignments from our PRIMe database. Purple ID s were annotated manually according to assignments in Figure S3.
