# Supplementary material for: Correlation exploration of metabolic and genomic diversity in rice
Source: BMC Genomics. 2009 Dec 1;10:568. doi: 10.1186/1471-2164-10-568 (PMC3087559; doi:10.1186/1471-2164-10-568)
Supplement: Additional file 9 — Figure S5. The correlative local genetic and metabolo-phenotypic diversity in rice. [file 1471-2164-10-568-S9.PDF]

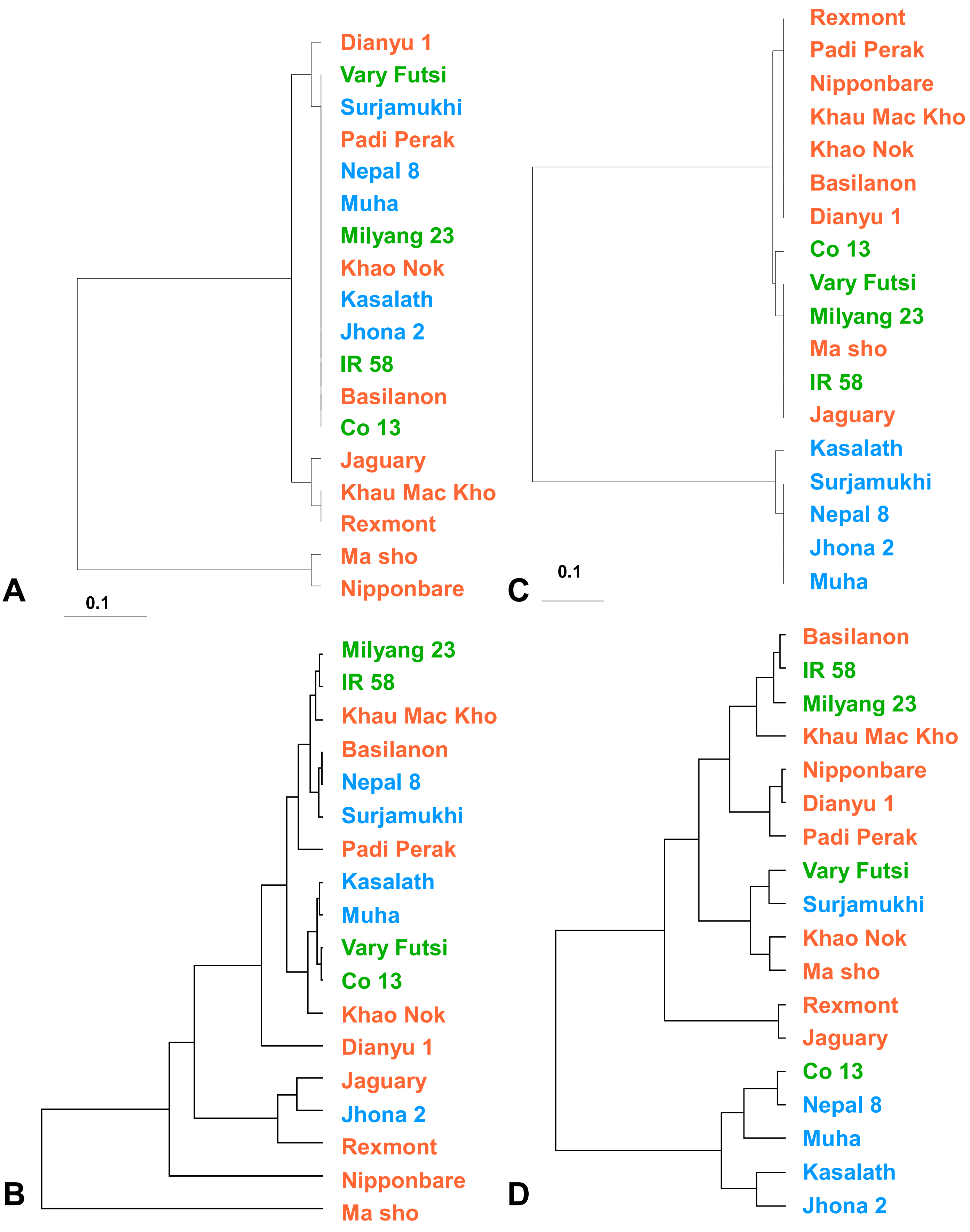

Figure. S5. Correlative local genetic and metabolo-phenotypic diversities in rice.

Dendrograms of phylogenetic relationships and of diversity of metabolic phenotypes, based on RFLP markers located on specific chromosomal regions and on 1D NMR spectral data for specific 1H chemical shift integral regions. (A, B) Genetic and metabolomic diversities corresponding to the chromosomal region including RFLP markers R1613, R1944 and G317 and the integral region of 1H chemical shift at 3.204 ppm of the NMR spectral data showed significant correlation (rs = 0.67). (C, D) The genetic polymorphisms in RFLP markers C43, G81 and C246 and the metabolic spectral diversity at 1.22 ppm, which corresponds to lipid metabolites containing -CH2- chains, also showed significant correlation (rs = 0.66).
