# Supplementary material for: Correlation exploration of metabolic and genomic diversity in rice
Source: BMC Genomics. 2009 Dec 1;10:568. doi: 10.1186/1471-2164-10-568 (PMC3087559; doi:10.1186/1471-2164-10-568)
Supplement: Additional file 10 — Figure S6. The correlative chromosomal regions for each NMR spectrum allocated on an RFLP map of rice. [file 1471-2164-10-568-S10.PDF]

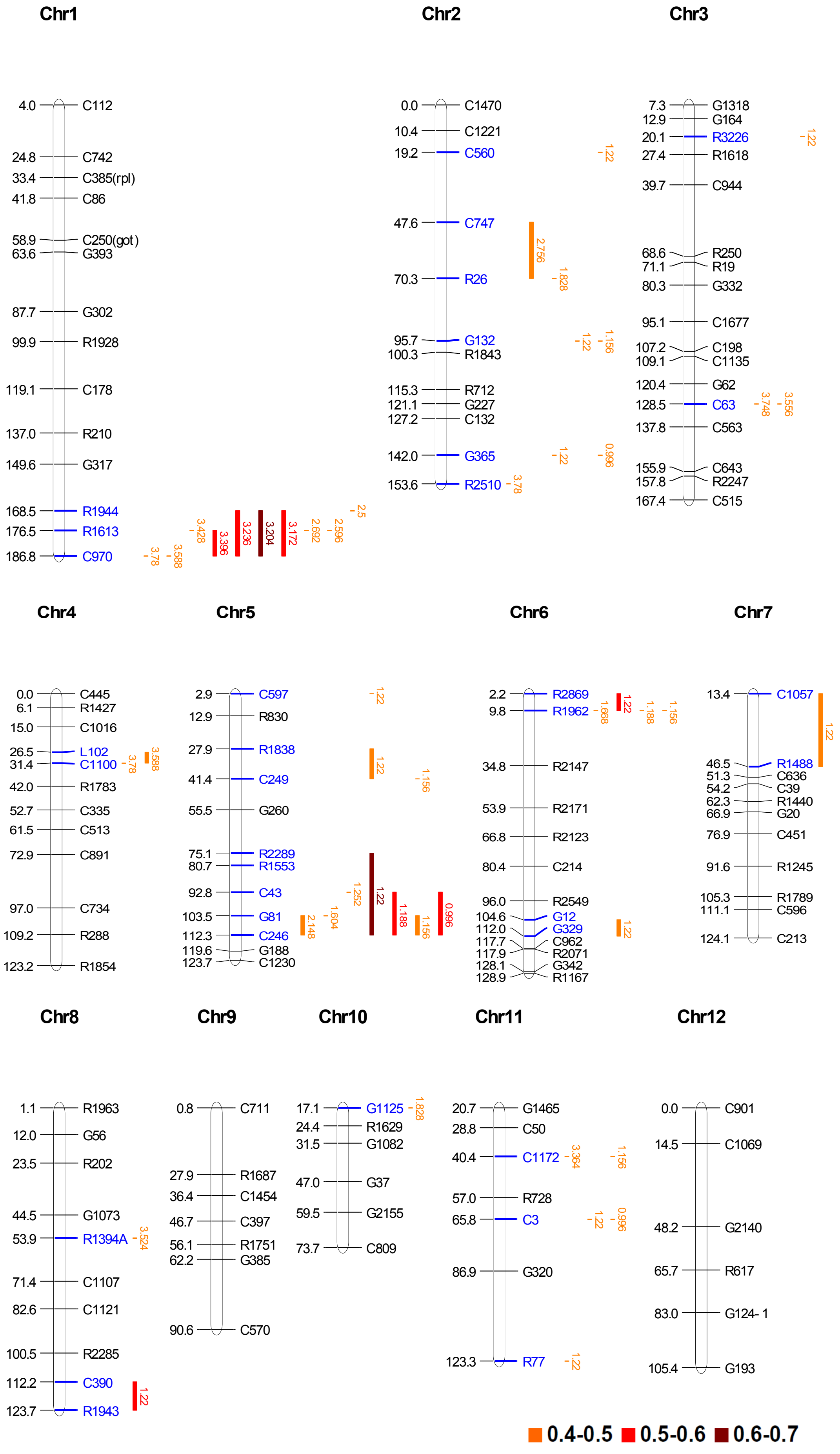

Figure S6. The correlative chromosomal regions for each NMR spectrum allocated on an RFLP map of rice.
