# Supplementary material for: Correlation exploration of metabolic and genomic diversity in rice
Source: BMC Genomics. 2009 Dec 1;10:568. doi: 10.1186/1471-2164-10-568 (PMC3087559; doi:10.1186/1471-2164-10-568)
Supplement: Additional file 11 — Figure S7. A schematic representation of the work flow used to explore the correlations between metabolic and genomic diversity with genetic resources. [file 1471-2164-10-568-S11.PDF]

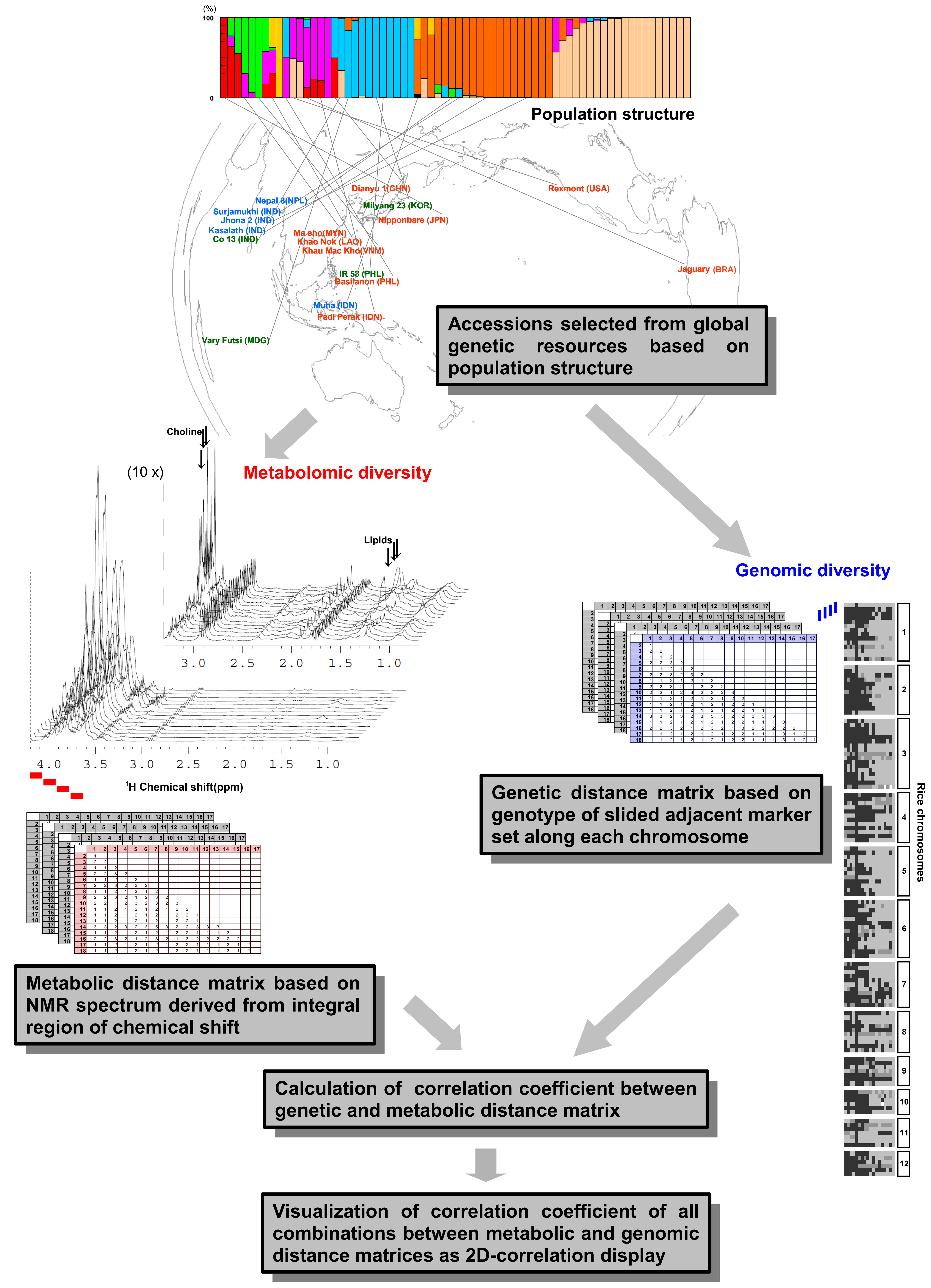

Figure S7. Schematic representation of the work flow to mine correlations between metabolic and genomic diversity in genetic resources.
