# Supplementary material for: Correlation exploration of metabolic and genomic diversity in rice
Source: BMC Genomics. 2009 Dec 1;10:568. doi: 10.1186/1471-2164-10-568 (PMC3087559; doi:10.1186/1471-2164-10-568)
Supplement: Additional file 7 — Figure S3. The 13C-HSQC spectra of seed extracts from the japonica and indica rice varieties. [file 1471-2164-10-568-S7.PDF]

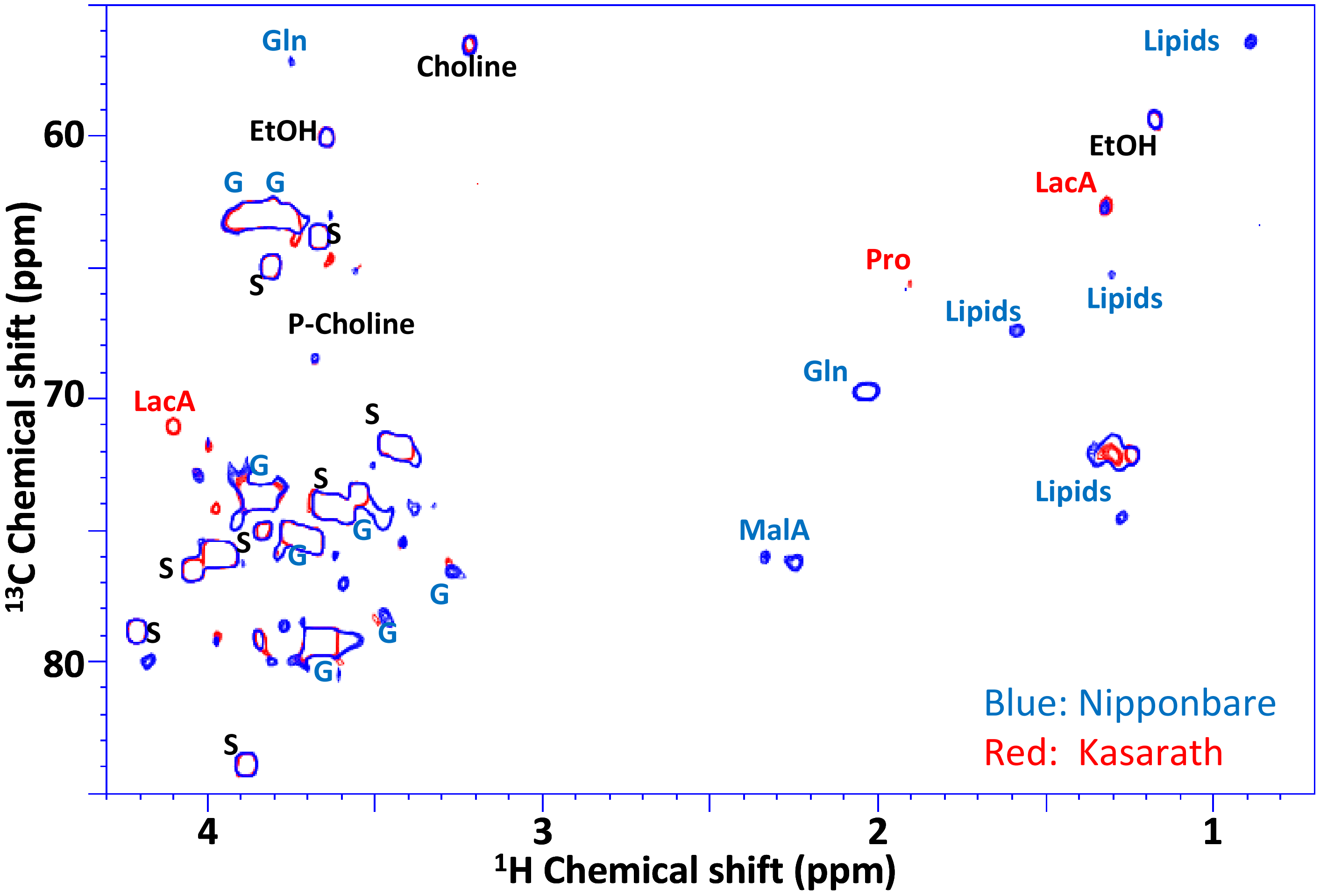

Figure. S3. 13C-HSQC spectra of seed extracts from japonica and indica rice varieties.
The different abundances of compounds in soluble seed extracts are shown by using 1H-NMR and 2D 13C-HSQC analyses of Nipponbare (japonica type, blue) and Kasalath (indica type, red). Signal assignments were based on intra-molecular spin–spin coupling correlations in 2D 1H–1H TOCSY spectra (data not shown).
